# Evaluation of the Clinical Rule for Endocarditis in the Emergency Department Among Patients With Suspected Infective Endocarditis

**DOI:** 10.1161/JAHA.123.032745

**Published:** 2024-02-14

**Authors:** Matthaios Papadimitriou‐Olivgeris, Pierre Monney, Pierre‐Nicolas Carron, Georgios Tzimas, Nicolas Beysard, Piergiorgio Tozzi, Matthias Kirsch, Benoit Guery

**Affiliations:** ^1^ Infectious Diseases Service Lausanne University Hospital and University of Lausanne Lausanne Switzerland; ^2^ Department of Cardiology Lausanne University Hospital and University of Lausanne Lausanne Switzerland; ^3^ Emergency Department Lausanne University Hospital and University of Lausanne Lausanne Switzerland; ^4^ Department of Cardiac Surgery Lausanne University Hospital and University of Lausanne Lausanne Switzerland

**Keywords:** clinical prediction rule, CREED score, emergency department, infective endocarditis, Infectious Endocarditis

Diagnosing infective endocarditis (IE) is challenging due to its variable clinical presentation, with patients often exhibiting nonspecific symptoms, requiring physicians in the emergency department (ED) to maintain high clinical suspicion.^1^ The Clinical Rule for Endocarditis in the ED (CREED) score demonstrates promising performance in diagnosing IE.^2^

This study aims to validate the score in a more specific population presenting a real clinical suspicion of IE. The data that support the findings of this study are available from the corresponding author upon reasonable request.

This study was conducted at the Lausanne University Hospital, Lausanne, Switzerland, from January 2014 to June 2023 (2014–2017: retrospective cohort of patients with IE, 2018–2023: prospective cohort of patients with suspected IE) (blood cultures drawn and echocardiography performed). The study was approved by the Swiss ethics committee (CER‐VD 2017–02137), and informed consent was obtained in the prospective cohort.

Inclusion criteria for this study were adult patients (≥18 years old) admitted to the ED with a temperature >37.5 °C (during the ED stay or 24 hours before admission). Patients whose initial symptoms developed after their ED stay were excluded.

Data on demographics, comorbidities, symptoms, physical signs, and laboratory values were collected from patients' electronic health records. A case was characterized as IE by the endocarditis team at day 60. SPSS version 26.0 (IBM, Armonk, NY) software was used for data analysis. Categorical variables were analyzed using the χ^2^ or Fisher exact test and continuous variables with the Mann‐Whitney *U* test. Bivariate analysis was performed with the dependent variable being IE. CREED score was calculated, and patients were divided in 4 risk groups (very low, low, high, and very high risk).^2^ Sensitivity, specificity, positive and negative predictive values, and 95% CIs were calculated using the predefined cutoff of +2 points.^2^ All statistic tests were 2‐tailed, and *P*<0.05 was considered statistically significant.

Among the 1749 episodes included in the cohort of suspected IE, 1137 (65%) were included; 394 patients whose initial symptoms developed after the ED stay and 218 with temperature ≤37.5 °C (of which 69 had IE) were excluded. In total, 473 (42%) patients had IE, 329 (70%) native and 127 (27%) prosthetic valve IE, and 47 (10%) cardiovascular implantable electronic device‐lead IE.

The Table depicts the characteristics of patients with and without final IE diagnosis by focusing on variables incorporated in the CREED score; 340 (30%), 227 (20%), 78 (7%), and 492 (43%) were classified as very low, low, high and very high‐risk groups, respectively. Within these categories, 42 (12%), 71 (31%), 43 (55%), and 317 (64%), respectively, had IE. By using the +2 points cutoff, sensitivity, specificity, positive predictive value, and negative predictive value were 91% (88%–94%), 45% (41%–49%), 54% (52%–56%), 88% (84%–91%), respectively.

**Table 1:** Characteristics of Patients With and Without Infective Endocarditis.

Characteristic	Without infective endocarditis (n=664)		Infective endocarditis (n=473)		*P* value
Demographics					
Male sex (+1 point)*	447	67%	360	76%	0.001
Age, y	67	55–77	67	51–77	0.182
Age ≥65 y	365	55%	260	55%	1.000
Comorbidities					
Charlson Comorbidity Index	4	2–7	4	1–6	<0.001
Congestive heart failure	54	8%	43	9%	0.591
Cirrhosis	53	8%	25	5%	0.095
Diabetes	158	24%	109	23%	0.777
Chronic kidney disease (moderate or severe)	154	23%	79	17%	0.009
Hemodialysis (+1 point)*	241	36%	92	20%	<0.001
Immunosuppression or active malignancy	241	36%	92	20%	<0.001
Malignancy (solid organ or hematological)	146	22%	43	9%	<0.001
Transplantation (solid organ or bone marrow)	28	4%	9	2%	0.040
HIV infection	29	4%	17	4%	0.545
Immunosuppressive treatment	133	20%	47	10%	<0.001
Prior hospitalization (within 1 mo) (+1 point)*	163	25%	70	15%	<0.001
Cardiac predisposing factors					
IV drug use	37	6%	45	10%	0.014
Prior endocarditis (+2 points)*	21	3%	48	10%	<0.001
Moderate/severe valvular disease (+2 points)*	44	7%	77	16%	<0.001
Prosthetic valve (+4 points)*	54	8%	147	31%	<0.001
Cardiac implantable electronic devices (+1 point)*	46	7%	83	18%	<0.001
Manifestations/clinical signs					
Malaise/asthenia	569	86%	453	96%	<0.001
Weight loss	40	6%	93	20%	<0.001
Dyspnea	128	19%	152	32%	<0.001
Thoracic pain (+1 point)*	32	5%	41	9%	0.010
Acute heart failure	49	7%	100	21%	<0.001
Neurological symptoms	108	16%	165	35%	<0.001
Temperature ≥39 °C	210	32%	149	32%	1.000
Sepsis	202	30%	206	44%	<0.001
Stroke (within 1 mo) (+3 points)*	27	4%	92	20%	<0.001
Physical signs of probable endocarditis (+11 points)*	167	25%	293	62%	<0.001
New heart murmur	111	17%	213	45%	<0.001
Embolic events (other than stroke)	61	9%	155	33%	<0.001
Osler nodes	1	0.2%	11	2%	<0.001
Imaging studies (within 24 h from emergency department admission)					
Cardiac echocardiography	109	16%	207	44%	<0.001
Thoracoabdominal CT scan	270	41%	201	43%	0.542
Cerebral imaging study (CT or MRI)	75	11%	131	28%	<0.001
Laboratory					
Anemia (+2 points)*	424	64%	298	63%	0.803
Leukocytosis	322	49%	231	49%	0.952
C‐reactive protein, mg/L (n=1078 patients)	138	67–243	162	83–262	0.005
Infectious diagnoses	490	74%	473	100%	<0.001
Pneumonia	56	8%	3	0.6%	<0.001
Urinary tract infection	52	8%	1	0.2%	<0.001
Abdominal infection	39	6%	1	0.2%	<0.001
Skin and soft tissue infection	104	16%	22	5%	<0.001
Bacteremia/candidemia	410	62%	439	93%	<0.001
Ascertained infective diagnosis (pneumonia, abdominal, urinary tract or skin and soft tissue infection) (−2 points)*	247	37%	27	6%	<0.001
CREED score	3	1–9	13	6–16	<0.001
CREED groups					
Very low risk (−2 to +2)	298	45%	42	9%	<0.001
Low risk (+3 to +5)	156	24%	71	15%	
High risk (+6 to +8)	35	5%	43	9%	
Very high risk (+9 to +24)	175	26%	317	67%	

Data are presented as number and percentage or median and quartile 1–quartile 3. CREED indicates Clinical Rule for Endocarditis in the Emergency Department; CT, computed tomography; and MRI, magnetic resonance imaging.

* Characteristics that are included in CREED score.

In our study, we sought to assess the usefulness in the ED of the CREED score among patients presenting with suspected IE. The CREED score, initially promising, did not perform as well, as expected, when applied to our specific patient population.^2^ One fundamental issue of the original study, as acknowledged by the authors, was the cohort's composition, with a staggering 87% inclusion classified as very low risk, many of whom had no true suspicion of IE, leading to an overestimation of the specificity and negative predictive value.^2^ Consequently, its application in the ED among patients with suspected IE should be limited.

Another limitation of the original study was the exclusion of patients with a temperature ≤37.5 °C. This criterion in the present study led to the exclusion of 218 episodes, including 69 IE cases. Although fever is a prominent IE symptom, its absence should not be used as a reason to exclude IE.^1^, ^3^

Furthermore, certain variables incorporated into the CREED score failed to effectively discriminate between patients with and without IE in our cohort. Notably, hemodialysis and recent hospitalization were more common in patients without IE, and anemia was consistent in both groups. These findings raise concerns about their relevance, particularly when considering that both variables are predominately associated with less common health care‐associated IE, rather than community‐acquired IE.^4^

Our study reaffirmed that certain factors, such as embolic events and various cardiac predisposing factors, are robust IE indicators.^4^, ^5^ Recognizing these factors should heighten suspicion among ED clinicians, especially in febrile patients, and prompt a thorough investigation for IE.

Nonetheless, our study has limitations. It was a single‐center study with some patients included retrospectively and a population that differs from that of the original study. We focused exclusively on patients with IE suspicion, unlike the original study that included all febrile patients. More studies are needed to evaluate the score's performance in the originally intended population.

In conclusion, our evaluation of the CREED score for diagnosing IE in the ED among patients with suspected IE revealed limitations, primarily due to the characteristics of the tested population. Future research should prioritize developing a more effective score to identify low‐risk patients, reducing the need for routine echocardiography.

## Sources of Funding

None.

## Disclosures

None.
